# Supplementary material for: CD20 Expression as a Possible Novel Prognostic Marker in CLL: Application of EuroFlow Standardization Technique and Normalization Procedures in Flow Cytometric Expression Analysis
Source: Cancers (Basel). 2022 Oct 7;14(19):4917. doi: 10.3390/cancers14194917 (PMC9562902; doi:10.3390/cancers14194917)
Supplement: Supplementary file 1 [file cancers-14-04917-s001.zip › cancers-1895762-supplementary.pdf]

Supplementary Material

CD20 expression as a possible novel prognostic marker in CLL: application of Euroflow standardization technique and normalization procedures in flow cytometric expression analysis

Anke Schilhabel[1], Peter Jonas Walter[1], Paula Cramer[2], Julia von Tresckow[3], Saskia Kohlscheen[1], Monika Szczepanowski[1], Anna Laqua[1], Kirsten Fischer[2], Barbara Eichhorst[2], Sebastian Böttcher[4], Christof Schneider[5], Eugen Tausch[5], Monika Brüggemann[1], Michael Kneba[1], Michael Hallek[2], Matthias Ritgen[1*]

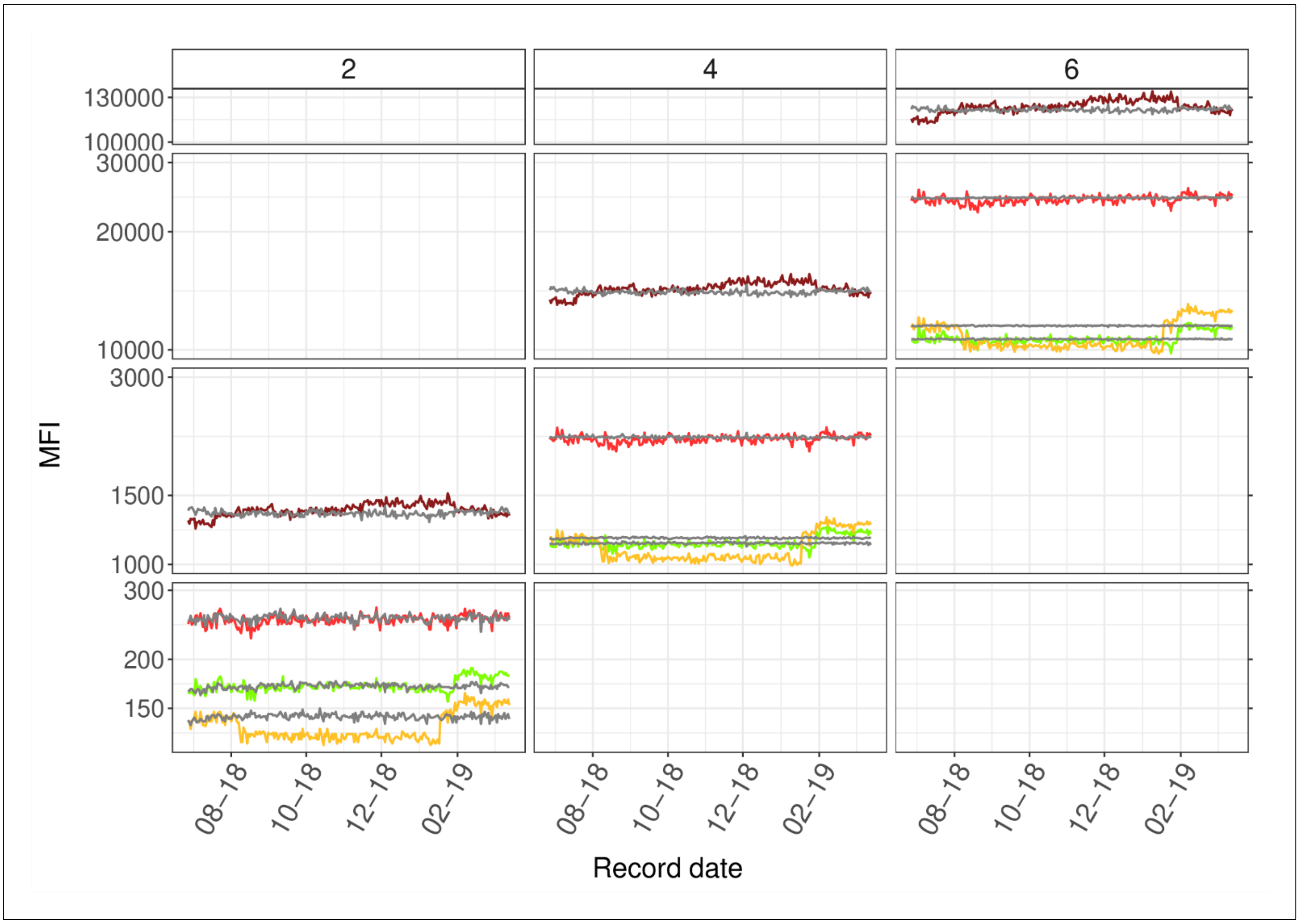

Figure S1 Normalization (grey) using the ratio of the measured MFI to the target value of the individual channels (FITC, PE, PerCP, APC) enables smoothing of MFI measurements across the MFI range provided by different peaks of theRainbow beads (2, 4, and 6 exemplarily shown).

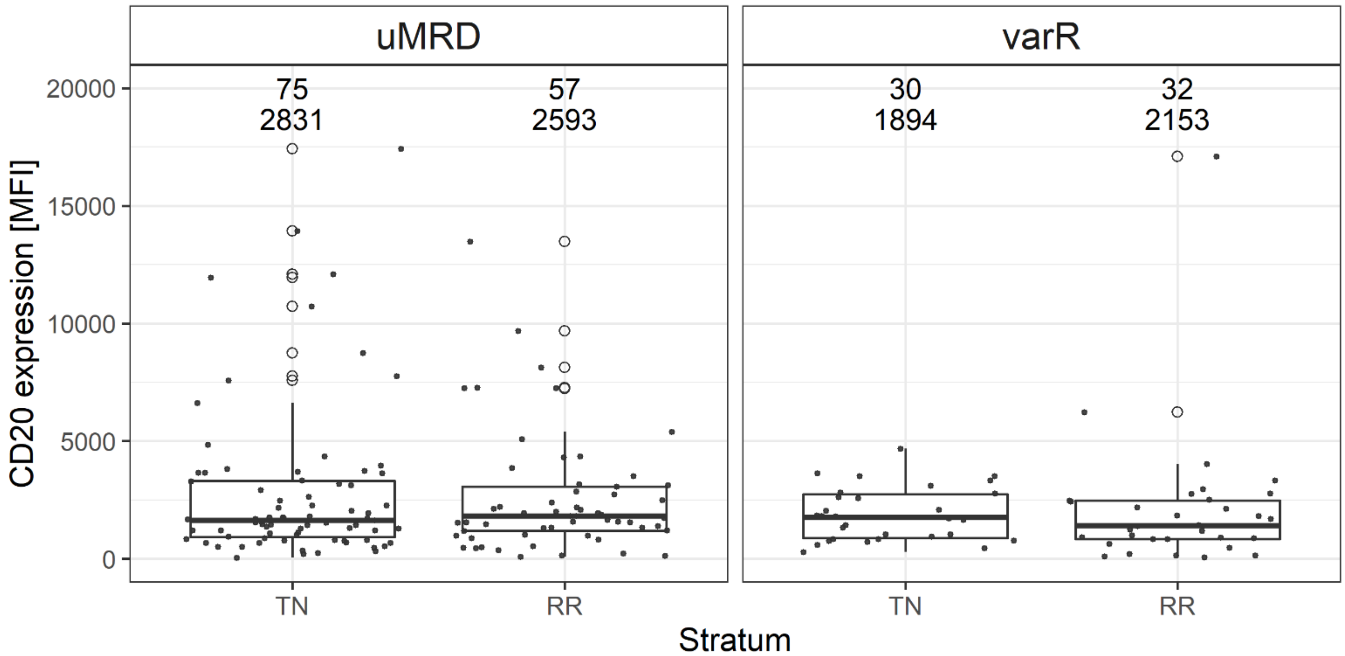

Figure S2 CD20 expression at the time point before therapy start in treatment naïve (TN) and relapsed/refractory (RR) patients categorized to different MRD response groups. Patients across all four CLL2-BXX trials were categorized in the groups achieving either sustained uMRD response or variable MRD response (varR: intermediate (IR), limited (LR) or no MRD response (NR)). Differences between groups were not statistically significant using Dunns test. The number of observations and the group mean CD20 MFI values are given above the plots.

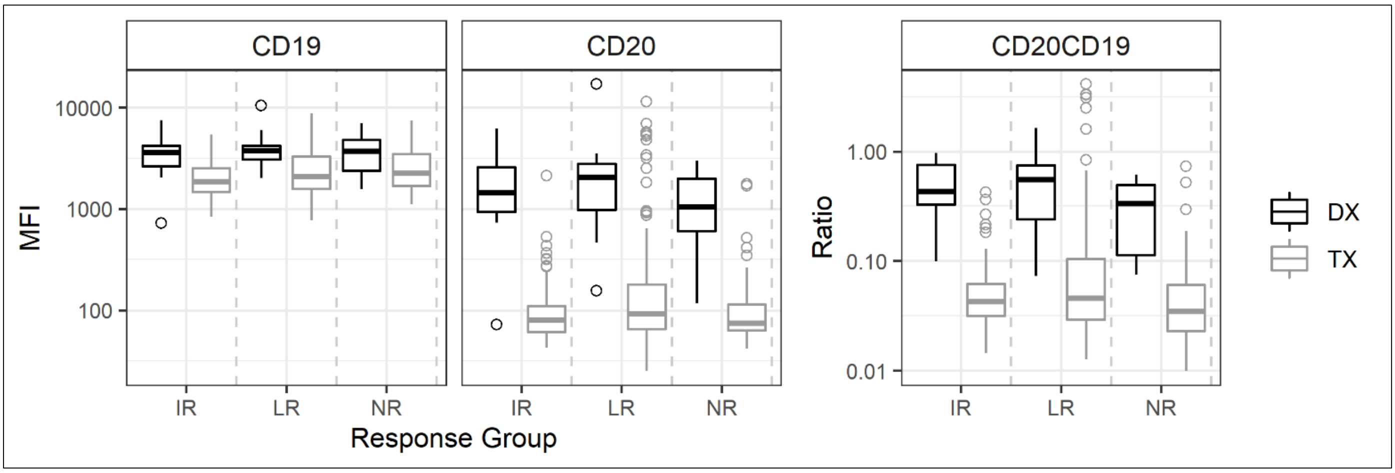

Figure S3 Expression of CD20 and CD19 (MFI) and CD20CD19 ratio of CLL cells in diagnostic (DX) and on-therapy (TX) samples for patients showing variable MRD response. Patients with intermediate (IR), limited (LR) response, and non-Responders (NR) were combined in the groups from the different CLL2-BXX trials. TX samples include time points from final restaging and different maintenance cycles. Differences between MRD response groups IR and LR were statistically significant for CD20 MFI (p=0.014) and the resulting CD20CD19 ratio (p=0.021).

Supplementary Material

Table S1 Patient characteristics in different response groups. BCG, BAG, BIG, BIO: patients with deep (uMRD) MRD response under the respective trial schemes (B: Bendamustine; I: Ibrutinib; A: Venetoclax; C: Idelalisib; G: Obinutuzumab; O: Ofatumumab), IR: intermediate MRD response; LR: limited MRD response; NR: Non-Responders; IR/LR/NR include patients from the different trial schemes

	BAG	BCG	BIG	BIO	IR	LR	NR	BIG_IR/LR	BIO_IR/LR	BIO_NR
N	55	16	46	15	25	23	14	10	30	13
age (mean, yr)	60.8	67.9	65.8	59.2	67.3	56.6	65.5	65.7	59.8	65.6
gender										
female/male	11/44	4/12	21/25	8/7	8/17	5/18	6/8	2/8	10/20	5/8
(%)	(20/80)	(25/75)	(45.7/54.3)	(53.3/46.7)	(32/68)	(21.7/78.3)	(42.9/57.1)	(20/80)	(33.3/66.7)	(38.5/61.5)
Binet stage										
A/(B/C)	14/41	4/12	12/34	7/8	3/22	5/18	2/12	1/9	6/24	1/12
(%)	(25.5/74.5)	(25/75)	(26.1/73.9)	(46.7/53.3)	(12/88)	(21.7/78.3)	(14.3/85.7)	(10/90)	(20/80)	(7.7/92.3)
Stratum										
TN/RR	33/22	6/10	27/19	9/6	14/11	10/13	6/8	2/8	21/9	6/7
(%)	(60/40)	(37.5/62.5)	(58.7/41.3)	(66.7/33.3)	(52/48)	(43.5/56.5)	(42.9/57.1)	(20/80)	(70/30)	(46,2/53.2)
TP53										
mutated/not mutated	13/42	5/11	8/38	2/13	11/14	9/14	5/9	3/7	11/19	4/9
(%)	(23.6/76.4)	(31.3/68.7)	(17.4/82.6)	(6.7/93.3)	(48/52)	(39.1/60.9)	(35.7/64.3)	(30/70)	(36.7/63.3)	(30.8/69.2)
IGHV										
mutated/not mutated/unknown	15/40	8/8	14/31/1	6/9	7/18	4/19	4/10	2/8	8/22	4/9
(%)	(27.3/72.7)	(50/50)	(30.4/67.4/2.2)	(40/60)	(28/72)	(17.4/82.6)	(28.6/71.4)	(20/80)	(26.7/73.3)	(30.8/69.2)
NOTCH1										
mutated/not mutated	6/49	3/13	9/37	4/11	3/22	4/19	3/11	2/8	4/26	2/11
(%)	(10.9/89.1)	(18.8/81.2)	(19.6/80.4)	(26.7/73.3)	(12/88)	(17.4/82.6)	(21.4/78.6)	(20/80)	(13.3/86.7)	(15.4/84.6)
Trisomy 12										
yes/no	9/46	2/14	8/38	6/9	4/21	4/19	3/11	2/8	5/25	2/11
(%)	(16.4/83.6)	(12.5/87.5)	(17.4/82.6)	(40/60)	(16/84)	(17.4/82.6)	(21.4/78.6)	(20/80)	(16.7/83.3)	(15.4/84.6)

TN treatment naïve; RR relapsed refractory

Supplementary Material

Table S2 Reduction of coefficients of variation for MFIs (CVmeasured/CVnormalized =CV/CV_n) of daily Rainbow bead controls by normalization (suffix a and b refer to the different CantoII instruments)

Peak	FITCa	PEa	PerCPa	APCa	FITCb	PEb	PerCPb	APCb	mean CV/CV_n	$SD_{CV/CVn}$	CV
2	2	4.4	1.9	2.1	1.9	1.5	1.2	1.6	1.74	0.30	17.04
3	3.1	10.2	3.2	2.6	3.9	3.8	2.1	1.8	2.93	0.74	25.40
4	5.4	20.1	4.6	2.8	7	7.5	3.2	1.9	4.63	1.97	42.66
5	7.9	30.4	7.3	2.9	10.4	10.4	5	1.9	6.54	3.15	48.12
6	11.7	32.6	9.1	3.8	13.9	10.2	4.3	2.2	7.89	4.13	52.38

Table S3 CD20 expression (MFI) in treatment naïve and relapsed/refractory patients in different response subgroups (varR: intermediate (IR), limited (LR) or no MRD response (NR), uMRD patients with deep MRD response splitted according to their treatment scheme (BCG, BAG, BIG, BIO B: Bendamustine; I: Ibrutinib; A: Venetoclax; C: Idelalisib; G: Obinutuzumab; O: Ofatumumab).

		Number of patients		Mean ± SD		Median	
		TN	RR	TN	RR	TN	RR
uMRD	BCG	6	10	1582±1627	2042±1142	862	1822
	BAG	33	22	2358±2664	2106±1872	1429	1438
	BIG	27	19	2500±2694	2470±2437	1697	1671
	BIO	9	6	6387±5768	5686±4741	3754	3116
varR	IR	14	11	2093±1256	1892±1783	1762	1183
	LR	10	13	2132±1017	2797±4424	2342	1816
	NR	6	8	1033±692	1465±1066	772	1552
